# Model-based forecasting for Canadian COVID-19 data

**DOI:** 10.1371/journal.pone.0244536

**Published:** 2021-01-19

**Authors:** Li-Pang Chen, Qihuang Zhang, Grace Y. Yi, Wenqing He

**Affiliations:** 1 Department of Statistical and Actuarial Sciences, University of Western Ontario, London, Ontario, Canada; 2 Department of Computer Science, University of Western Ontario, London, Ontario, Canada; Rutgers University/New Brunswick, UNITED STATES

## Abstract

**Background:**

Since March 11, 2020 when the World Health Organization (WHO) declared the COVID-19 pandemic, the number of infected cases, the number of deaths, and the number of affected countries have climbed rapidly. To understand the impact of COVID-19 on public health, many studies have been conducted for various countries. To complement the available work, in this article we examine Canadian COVID-19 data for the period of March 18, 2020 to August 16, 2020 with the aim to forecast the dynamic trend in a short term.

**Method:**

We focus our attention on Canadian data and analyze the four provinces, Ontario, Alberta, British Columbia, and Quebec, which have the most severe situations in Canada. To build predictive models and conduct prediction, we employ three models, *smooth transition autoregressive* (STAR) models, *neural network* (NN) models, and *susceptible-infected-removed* (SIR) models, to fit time series data of confirmed cases in the four provinces separately. In comparison, we also analyze the data of daily infections in two states of USA, Texas and New York state, for the period of March 18, 2020 to August 16, 2020. We emphasize that different models make different assumptions which are basically difficult to validate. Yet invoking different models allows us to examine the data from different angles, thus, helping reveal the underlying trajectory of the development of COVID-19 in Canada.

**Finding:**

The examinations of the data dated from March 18, 2020 to August 11, 2020 show that the STAR, NN, and SIR models may output different results, though the differences are small in some cases. Prediction over a short term period incurs smaller prediction variability than over a long term period, as expected. The NN method tends to outperform other two methods. All the methods forecast an upward trend in all the four Canadian provinces for the period of August 12, 2020 to August 23, 2020, though the degree varies from method to method. This research offers model-based insights into the pandemic evolvement in Canada.

## 1 Introduction

Since the first case of the coronavirus disease 2019 (COVID-19) was found in Wuhan, China in December 2019, the disease has been spreading worldwide. In Canada, the first confirmed case appeared in the early January, and as of August 17, 2020, 122,392 cumulative confirmed cases have been reported and the pandemic does not seem to be over in the next short period. To assess the impact of COVID-19 in Canada, a large body of research has been done. For example, Tuite et al. [1] examined an age-structured compartmental model for COVID-19 transmissions in the population of Ontario, Canada. Doreleyers and Knighton [2] studied the dataset collected from over 100,000 postsecondary students from April 19, 2020 to May 1, 2020 and discussed how their academic life was impacted by the COVID-19 pandemic. Financial impacts on multiple perspectives, such as economic concerns of immigrants, work interruptions, or postsecondary students, were discussed by Wall [3], Messacar and Morissette [4], and LaRochelle-Côté and Uppal [5], among others. Information on various impacts of COVID-19 can be found in https://www.statcan.gc.ca/eng/covid19.

While different studies on COVID-19 become available, it is important to forecast the trajectories of the development of COVID-19. Model-based forecasting has been explored by various authors. For example, treating COVID-19 data as time series, Tandon et al. [6] and Bayyurt and Bayyurt [7] respectively applied autoregressive integrated moving average (ARIMA) models to predict the future infected cases and death. Petropoulos and Makridakis [8] employed the exponential smoothing method to model the cumulative number of infected cases. Siedner et al. [9] used time series methods to illustrate that social distancing helps slow down the COVID-19 epidemic in the U.S. On the other hand, epidemic modeling has also been broadly considered. For example, Fanalli and Piazza [10] implemented the susceptible-infected-removed (SIR) model to analyze and forecast the COVID-19 spread in China, Italy, and France. Wang et al. [11] extended the SIR models to evaluate the non-pharmaceutical intervention on the outbreak of COVID-19 in Wuhan, China.

While different models have been employed separately by different researchers to study the development trajectory of COVID-19, it is unclear how these models may perform because the associated model assumptions are typically untestable. In this paper we implement three prediction methods to study the COVID-19 data in Canada and compare their forecasting performance. Specifically, different from the past literature that directly used linear time series models to fit data, we consider nonlinear time series model, the *smooth transition autoregressive models* (STAR), as well as the machine learning method, the *neural network* (NN) model. Moreover, for the sake of comparison, we also apply the SIR model to characterize the trajectory of the number of infected cases.

To reflect possibly different measures taken by the local government in each province to curb the virus spread, our discussion is carried out separately for individual provinces, which involve Ontario, Alberta, British Columbia, and Quebec, the four provinces that have large numbers of infected cases. Our study is conducted for the dataset available in https://coronavirus.1point3acres.com/en.

## 2 Data and framework

### 2.1 Descriptive statistics

**Fig 1:**
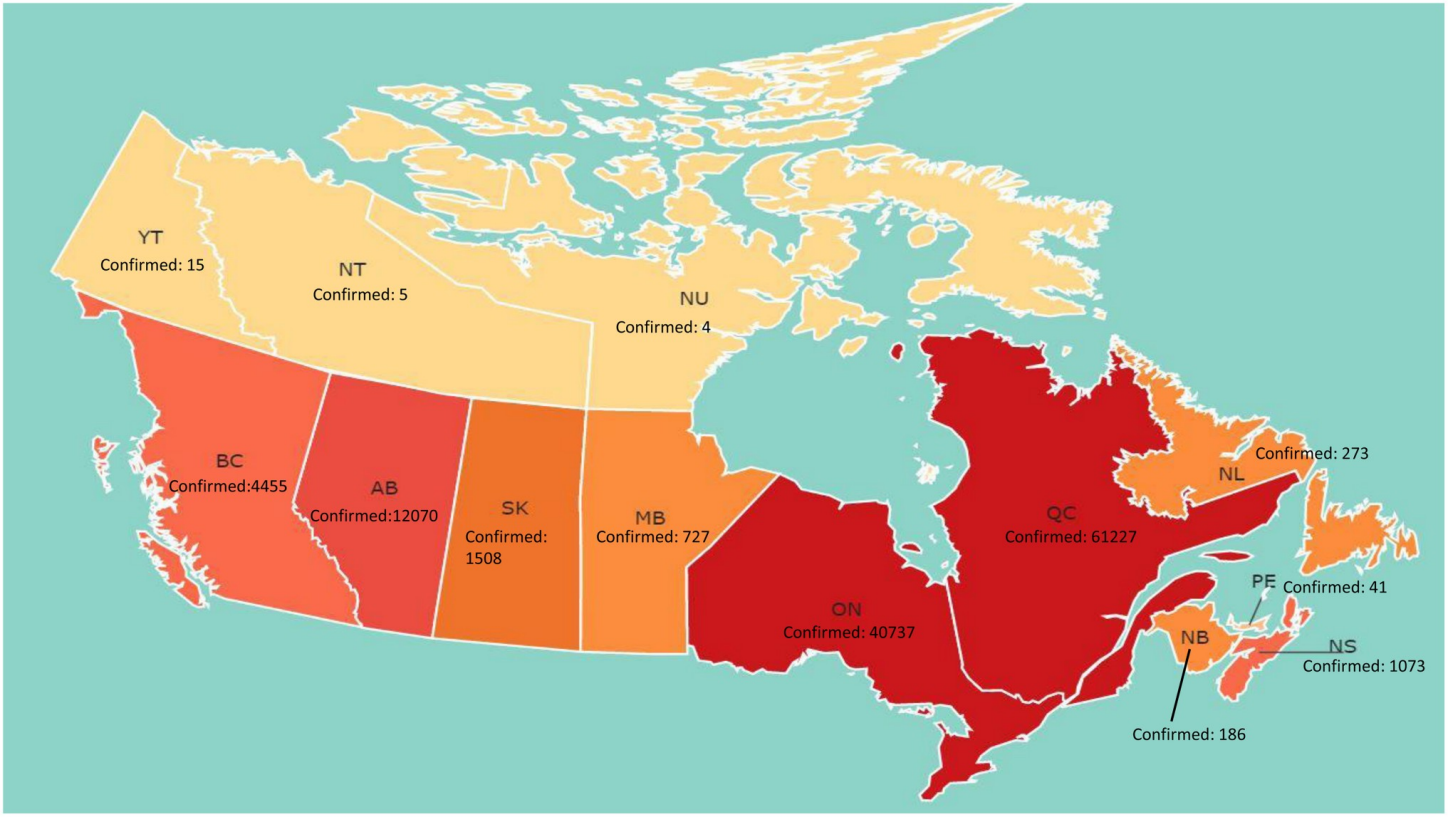
The number of cumulative confirmed cases with COVID-19 in each province of Canada as of August 16, 2020. Darker color indicates a higher number of the confirmed cases. Reprinted from https://covid-19-canada.uwo.ca/ under a CC BY license, with permission from the GW-DSRG (Grace-Wenqing Data Science Research Group), original copyright 2020.

**Fig 2:**
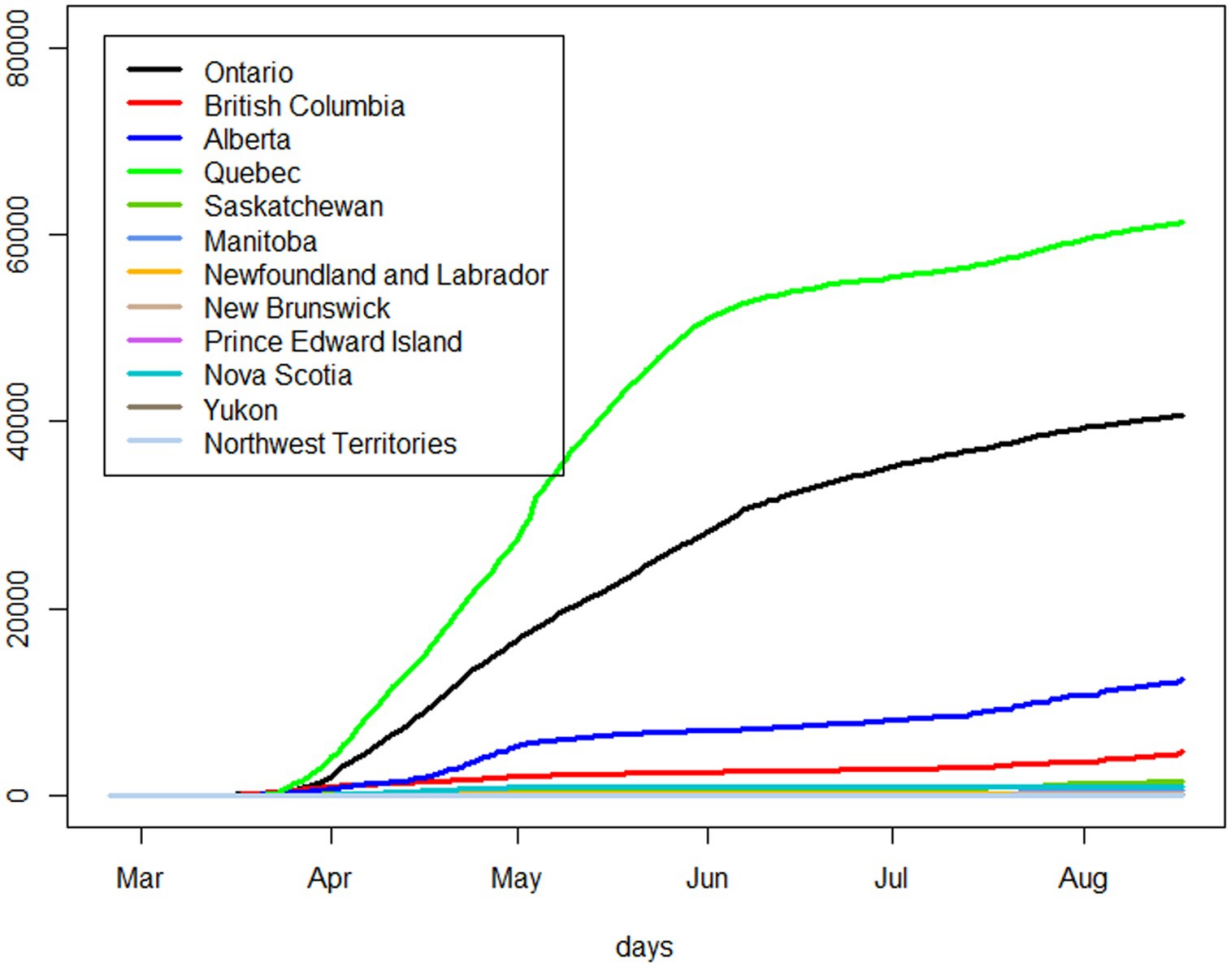
The cumulative number of infected cases with COVID-19 in Canada.

The dataset, dated from February 24, 2020 to August 16, 2020, is available at https://coronavirus.1point3acres.com/en. It records the number of infected cases and the number of deaths on a daily basis for each province or territory in Canada. Fig 1 gives a map display of the total number of infected cases for each province, and Fig 2 further displays the number of cumulative infected cases since February 24, 2020. Ontario and Quebec have the largest numbers of cumulative confirmed cases, and the second cluster of severely infected provinces includes Alberta and British Columbia. These four provinces are more populated than other Canadian provinces. To better show the relation between the population size and the number of cumulative confirmed cases, in Table 1 we report the infection rate as of August 16 which is defined as the ratio of the number of total infected cases to the population size for the four provinces as well as for the entire Canada. While Ontario is the most populated, its infection rate is not the highest and is even lower than the overall infection rate in Canada. More detailed explorations and descriptive statistics of the Canadian data are available in the dashboard created by the GW-DS-COVID-19 research group: https://covid-19-canada.uwo.ca/index.html which was introduced by Liu et al. [12].

**Table 1:** A comparison of the population size, the number of cumulative confirmed cases, and the infection rate in Canada and the four provinces.

Region	Number of total infected cases	Population size*	Infection rate(%)
Canada	122,392	37,314,442	0.328
Ontario	40,737	14,446,515	0.282
Quebec	61,227	8,433,301	0.726
Alberta	12,070	4,345,737	0.278
British Columbia	4,455	5,020,302	0.089

*Website source: https://worldpopulationreview.com/canadian-provinces/

**Fig 3:**
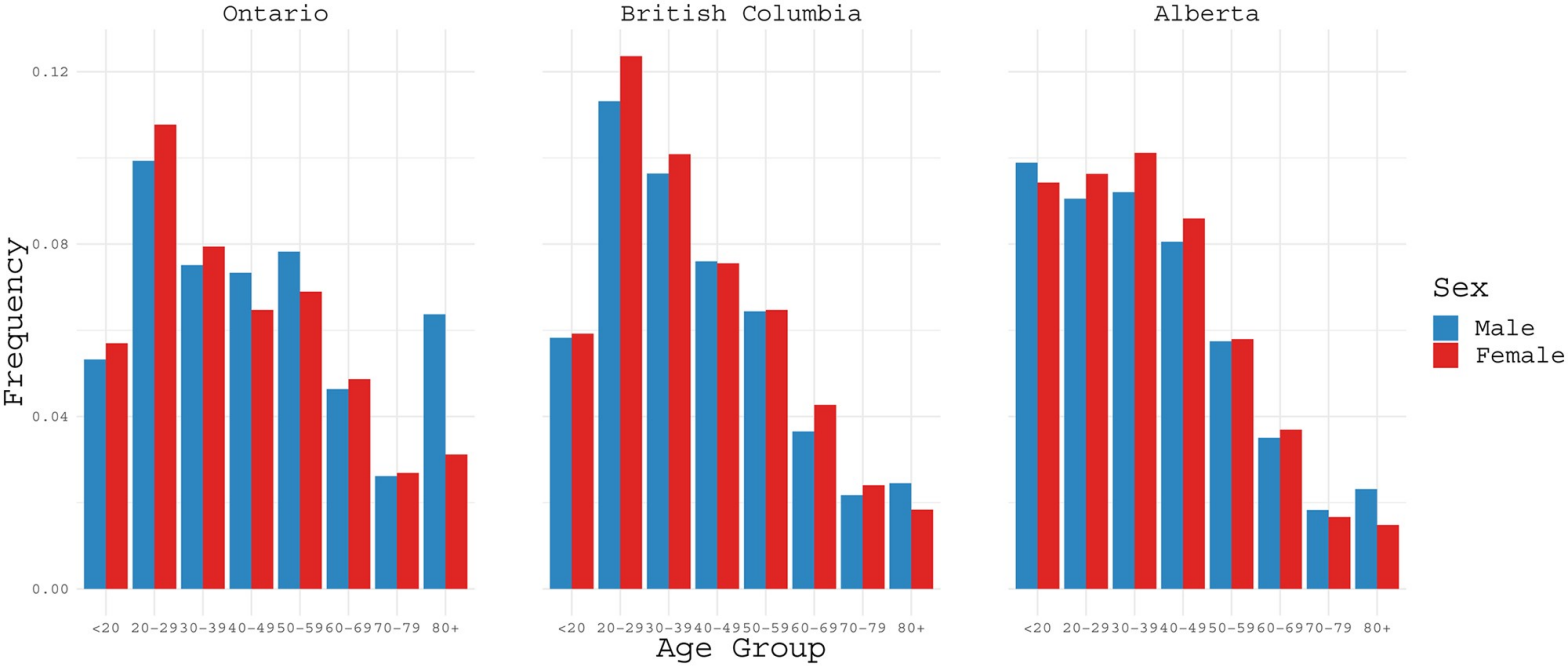
COVID-19 infection rates classified by age ranges (in years) for Ontario, British Columbia and Alberta based on the data as of August 16, 2020.

Fig 3 reports infection rates classified by age (in years) and gender for the provinces Ontario, British Columbia, and Alberta based on the data reported as of August 16, 2020. Infection rates seem to be fairly close for men and women in the same groups but differ noticeably for people at different ages. The highest infection rate is in the age interval 20-29 for Ontario and British Columbia, whereas infection rates in Alberta appear fairly similar for age up to 49. For the group of aged 80 and older, infection rates for men are higher than those for women, and particularly, the infection rate for men doubles that for women in Ontario.

**Fig 4:**
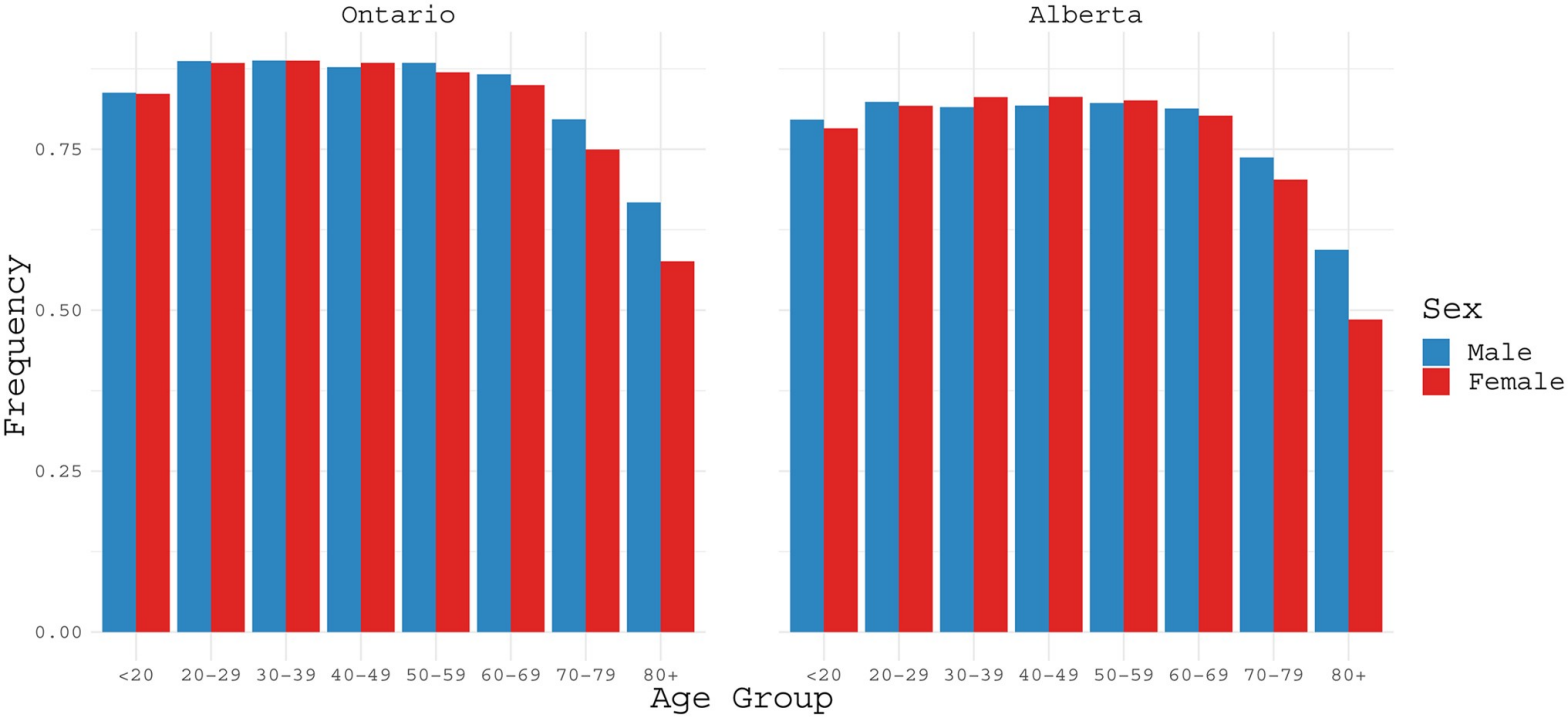
COVID-19 recovery rates classified by age ranges (in years) for Ontario and Alberta based on the data as of August 16, 2020.

Based on the available data, in Fig 4 we display recovery rates as of August 16, 2020, for Ontario and Alberta according to the information of age and gender. Recovery rates appear similar for men and women, and they remain fairly the same for different age groups except for individuals aged 70 or older. For this age group, recovery rates for males are higher than females. For patients younger than 70, recovery rates are higher than 80%.

### 2.2 Framework of time series analysis

Since most provinces in Canada declared “the state of emergency” as of March 18, 2020, we analyze time series data of the number of daily confirmed cases since March 18, 2020 which are likely to be more homogeneous than the data before this date. We are interested in forecasting the trend of the pandemic in each of the four provinces. Shown in Table 2, we take the dataset in the period from March 18, 2020 to August 11, 2020 as the training set to construct prediction models, and use the data from August 12, 2020 to August 16, 2020 as the testing data. The goal is to predict the number of cases in the “future” days, where we consider a short term period from August 12, 2020 to August 23, 2020 in which the testing data in the first five days can be used to assess the performance of prediction. In comparison, we also conduct prediction for a longer period of 25 days starting from August 12, 2020, though more variability is expected.

**Table 2:** Data split and prediction.

Type	Period	Number of days
Training Data	March 18 to August 11	147
Testing Data	August 12 to August 16	5
Short-term Prediction	August 12 to August 23	12
Long-term Prediction	August 12 to September 05	25

## 3 Methods and analysis results

To construct prediction models with time-dependent data, techniques of handling time series can be employed. To address the nonlinear patterns shown in Fig 2, we apply three modeling methods: *smooth transition autoregressive* (STAR) models, *neural network* (NN) models, and *susceptible-infected-removed* (SIR) models. We first describe these models, and then present the results for the COVID-19 data of the four Canadian provinces as well as two states in USA.

### 3.1 Modeling and prediction

#### 3.1.1 The STAR model

For the discrete time point *t* = 0, 1, 2, …, *T*, let *X*_*t*_ denote the random process of interest. The STAR model (e.g., Chatfield and Xing 2019 [13], Section 11.4) assumes the form

$$\begin{array}{c} X_{t}=(a_{0}+\sum_{j=1}^{p}a_{j}X_{t-j})\varphi (X_{t-d})+(b_{0}+\sum_{j=1}^{p}b_{j}X_{t-j})\{1-\varphi (X_{t-d})\}+\epsilon_{t}, \end{array}$$ (1)

where the *ϵ*_*t*_ are white noises which are assumed to be independent and identically distributed with mean zero. Here *a*_0_, *a*_*j*_, *b*_0_, and *b*_*j*_ are unknown parameters, *p* is an order of the autoregressive process, *d* is the delay parameter, and *φ*(⋅) is a smooth function taken as, for example, the logistic function with a parameter, say *α* (Chatfield and Xing 2019 [13], p.280), as in our numerical studies.

Let *θ* = (*a*_0_, *a*_1_, …, *a*_*p*_, *b*_0_, *b*_1_, …, *b*_*p*_, *α*)^⊤^ denote the vector of associated parameters. It can be estimated by the least squares method, given by

$$\begin{array}{c} \hat{\theta }=\underset{\theta }{\mathrm{argmin}}\sum_{t=1}^{T}{\left\{X_{t}-F\left(X_{t};\theta \right)\right\}}^{2} \end{array}$$ (2)

with $F\left(X_{t};\theta \right)=(a_{0}+\sum_{j=1}^{p}a_{j}X_{t-j})\varphi (X_{t-d})+(b_{0}+\sum_{j=1}^{p}b_{j}X_{t-j})\{1-\varphi (X_{t-d})\}$. Estimator $\hat{\theta }$ is equivalent to the maximum likelihood estimator if *ϵ*_*t*_ is further assumed to follow a normal distribution (van Dijk et al. 2002 [14], p.19).

To calculate a forecasted value, we first let ${\hat{X}}_{t}$ denote the fitted value for *t* = 1, …, *T*, obtained from (1) with *θ* replaced by (2), and let $e_{t}=X_{t}-{\hat{X}}_{t}$ denote the resulting residual. Then the residual standard deviation is defined as (e.g., Hyndman and Athanasopoulos 2018 [15], Section 5.2)

$$\begin{array}{c} \hat{\sigma }≜\sqrt{\frac{1}{T-1}\sum_{t=1}^{T}e_{t}^{2}}. \end{array}$$

Suppose we are interested in forecasting the value at time point *T* + *h*, where *h* represents the number of steps in prediction. Then we use (1) recursively to work out the predicted values ${\hat{X}}_{T+1},\ldots ,{\hat{X}}_{T+h}$, respectively, for time points *t* = *T* + 1, …, *T* + *h*, where *θ* is replaced by $\hat{\theta }$. Further, we construct the associated 95% as

$$\begin{array}{c} {\hat{X}}_{T+h}\pm 1.96{\hat{\sigma }}_{h}, \end{array}$$

where ${\hat{\sigma }}_{h}=\hat{\sigma }\sqrt{h}$ is the standard deviation of the *h*-step forecast (e.g., Hyndman and Athanasopoulos 2018 [15], Section 3.5). The increase of ${\hat{\sigma }}_{h}$ with *h* shows that the forecast becomes more variable for prediction at a longer time point.

The R functions lstar and forecast (Chatfield and Xing 2019 [13], p.281) can be used to fit the model using the training data and perform prediction by constructing 95% prediction intervals, respectively.

#### 3.1.2 The NN model

**Fig 5:**
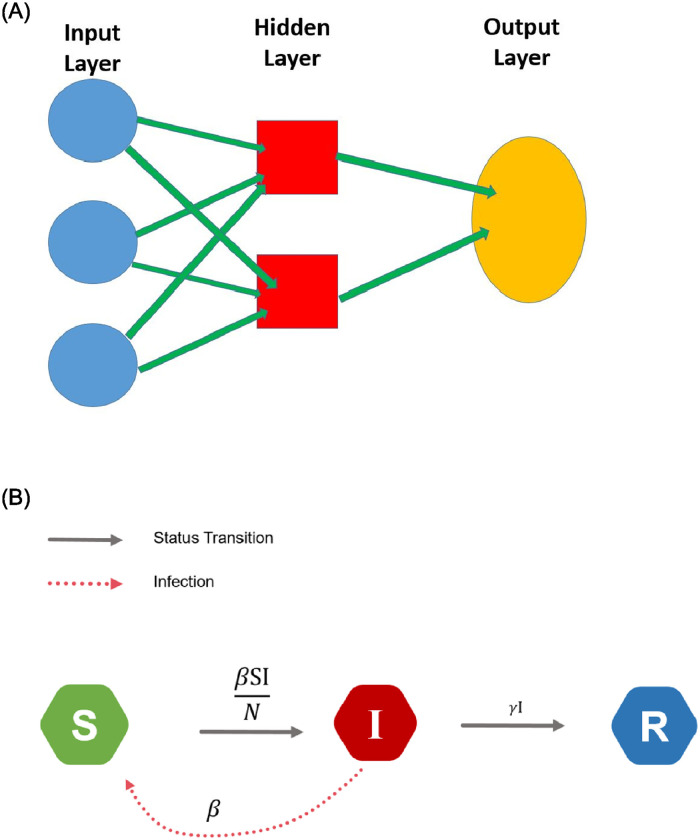
Illustration diagrams: The left panel is for the NN model, and the right panel is for the SIR model.

The *neural network* (NN) model is an important tool in machine learning, which basically includes three elements: the input layer, the hidden layer(s), and the output layer, as illustrated in the left panel of Fig 5. For example, consider the case with one hidden layer with *J* nodes. The *T* time series variables *X*_*t*_ are taken in the input layer, and the weighted sum for the *j*th node in the hidden layer is given by

$$\begin{array}{c} V_{j}=\sum_{t=1}^{T}w_{tj}X_{t} \end{array}$$

for *j* = 1, …, *J*, where *w*_*tj*_ are weights to be tuned. Then through activation functions, the value in the output layer is formulated as

$$\begin{array}{c} {\hat{X}}_{t}=\phi_{0}\{\sum_{j=1}^{J}w_{j}^{*}\phi_{h}\left(V_{j}\right)+w_{0}^{*}\}, \end{array}$$

where $w_{j}^{*}$ and $w_{0}^{*}$ are weights, and *φ*_*h*_(⋅) and *φ*_0_(⋅) are the user-specified *activation functions*. The weights are estimated by minimizing $\sum_{t=1}^{T}{\left({\hat{X}}_{t}-X_{t}\right)}^{2}$. Prediction of a future time point proceeds in the same way as that of the STAR model.

The R function nnetar can be used to fit the training data, where activation functions are respectively specified as

$$\begin{array}{c} \phi_{h}\left(v\right)={\left\{1+\exp(-v)\right\}}^{-1}\;\;\text{and}\;\;\phi_{0}\left(v\right)=v. \end{array}$$

The R function forecast can be invoked to produce predicted results as well as 95% prediction intervals (Chatfield and Xing 2019 [13], p.295). In our numerical studies, we take one hidden layer with *J* = 3 nodes.

#### 3.1.3 The SIR model

In contrast to the STAR and NN model which facilitate a stochastic variation (Chatfield and Xing 2019 [13], Section 3.1), the susceptible-infected-removed (SIR) model is a deterministic epidemic model. The STAR and NN models postulate the number of infected cases only, whereas the SIR model incorporates not only the infected cases but also the numbers of death and recovery. The SIR model basically employs differential equations to describe the dynamic changes of the population which is classified as three compartments: *susceptible* (S), *infected* (I) and *removed* (R) (consisting of recoveries and deaths).

The status for an individual in the population may change with time: a healthy individual may become infected, and an infected patient may recover or die of the disease, as shown in the right panel of Fig 5, where transition rates are introduced to characterize the dynamic changes. Let *S*_*t*_, *I*_*t*_, and *R*_*t*_, respectively, denote the size of the population in the states of susceptible, infected, and removed at time *t*, and let *N* ≜ *S_t_* + *I_t_* + *R_t_* denote the population size which is assumed to be fixed. Let *β* denote the average number of contacts per infectious person per time unit, and let *γ* represent the transition rate from *I*_*t*_ to *R*_*t*_; in other words, the duration of the infectious status is characterized by $\frac{1}{\gamma }$.

As a result, the SIR model is given by the ordinary differential equations:

$$\begin{array}{cc} \frac{dS_{t}}{dt} & =-\frac{\beta I_{t}S_{t}}{N};\\ \frac{dI_{t}}{dt} & =\frac{\beta I_{t}S_{t}}{N}-\gamma I_{t};\\ \frac{dR_{t}}{dt} & =\gamma I_{t}; \end{array}$$ (3)

where one equation is determined by other two equations due to the constraint that the total population size remains unchanged. The R function SIR in the EpiDynamics package can be implemented to simulate *I*_*t*_ and *R*_*t*_ from the differential (Eq 3).

While the SIR model is based on the modeling of *S*_*t*_, *I*_*t*_, and *R*_*t*_, our focus here concerns the daily infection numbers and aims to do prediction with minimal prediction errors. Regarding the daily number of confirmed cases on day *t* as the difference of *S*_*t*_ and *S*_*t*−1_, we calculate the predicted number of daily confirmed cases on day *t*, denoted ${\hat{X}}_{t}\left(\beta ,\gamma \right)$, as follows:

$$\begin{array}{ccc} {\hat{X}}_{t}\left(\beta ,\gamma \right) & = & S_{t-1}-S_{t}\\ & = & \left(N-I_{t-1}-R_{t-1}\right)-\left(N-I_{t}-R_{t}\right)\\ & = & I_{t}-I_{t-1}+R_{t}-R_{t-1} \end{array}$$

for *t* = 1, …, *T*, where *T* represents the end of the study period, the assumption of the fixed population size is used, and the dependence of *I*_*t*_, *I*_*t*−1_, *R*_*t*_, and *R*_*t*−1_ on *β* and *γ* is implicitly reflected from the system of the ordinary differential (Eq 3). Then the parameters *β* and *γ* in (3) can be obtained by minimizing the squared prediction error

$$\begin{array}{c} \text{PE}\left(\beta ,\gamma \right)=\sum_{t=1}^{T}{\left\{X_{t}-{\hat{X}}_{t}\left(\beta ,\gamma \right)\right\}}^{2} \end{array}$$ (4)

with respect to *β* and *γ*.

The minimization of (4) can be realized by using the R function optim in the built-in stats package. Prediction values and associated intervals can be computed following the same lines as discussed by Efimov and Ushirobira (2020) [16]. Specifically, let ${\hat{\sigma }}_{\beta }$ and ${\hat{\sigma }}_{\gamma }$ denote the estimated standard deviations of the estimators $\hat{\beta }$ and $\hat{\gamma }$, computed by applying the function optim in the built-in stats package via the gradient descent of (4). Therefore, the 95% confidence interval for $\hat{\beta }$ and $\hat{\gamma }$ are, respectively, given by $\left(\underline{\beta },\bar{\beta }\right)$ and $\left(\underline{\gamma },\bar{\gamma }\right)$, where

$$\begin{array}{c} \underline{\beta }=\hat{\beta }-1.96{\hat{\sigma }}_{\beta },\;\;\bar{\beta }=\hat{\beta }+1.96{\hat{\sigma }}_{\beta },\;\;\underline{\gamma }=\hat{\gamma }-1.96{\hat{\sigma }}_{\gamma },\;\;\text{and}\;\;\bar{\gamma }=\hat{\gamma }+1.96{\hat{\sigma }}_{\gamma }. \end{array}$$

Following Efimov and Ushirobira [16], we create the prediction bound in the following procedure:

Step 0: Initialize *S*_0_, *I*_0_ and *R*_0_. Set *t* = 0.
Step 1: Simulate a lower bound of *S*_*t*_, *I*_*t*_ and *R*_*t*_, denoted $\underline{S_{t}}$, $\underline{I_{t}}$ and $\underline{R_{t}}$, and an upper bound of *S*_*t*_, *I*_*t*_ and *R*_*t*_, denoted $\bar{S_{t}}$, $\bar{I_{t}}$ and $\bar{R_{t}}$ by
  $$\begin{array}{cc} {\underline{S}}_{t+1} & =\left(1-\frac{\bar{\beta }{\bar{I}}_{t}}{N}\right){\underline{S}}_{t};\\ {\underline{I}}_{t+1} & =\left(1-\bar{\gamma }\right){\underline{I}}_{t}+\underline{\beta }{\underline{I}}_{t}{\underline{S}}_{t};\\ {\underline{R}}_{t+1} & ={\underline{R}}_{t}+\underline{\gamma }{\underline{I}}_{t};\\ {\bar{S}}_{t+1} & =\left(1-\frac{\underline{\beta }{\underline{I}}_{t}}{N}\right){\bar{S}}_{t};\\ {\bar{I}}_{t+1} & =\left(1-\underline{\gamma }\right){\bar{I}}_{t}+\bar{\beta }{\bar{I}}_{t}{\bar{S}}_{t};\\ {\bar{R}}_{t+1} & ={\bar{R}}_{t}+\bar{\gamma }{\bar{I}}_{t}. \end{array}$$
Step 2: Calculate the upper bound ${\bar{\hat{X}}}_{t+1}$ and the lower bound ${\underline{\hat{X}}}_{t+1}$ of the 95% prediction interval for time point *t* + 1, given by ${\bar{\hat{X}}}_{t+1}={\bar{I}}_{t}-{\underline{I}}_{t-1}+{\bar{R}}_{t}-{\underline{R}}_{t-1}$ and ${\underline{\hat{X}}}_{t+1}={\underline{I}}_{t}-{\bar{I}}_{t-1}+{\underline{R}}_{t}-{\bar{R}}_{t-1}$.
Step 3: If *t* < *T*, then set *t* ≔ *t* + 1 and back to Step 1.

### 3.2 Analysis of the data in four Canadian provinces

**Fig 6:**
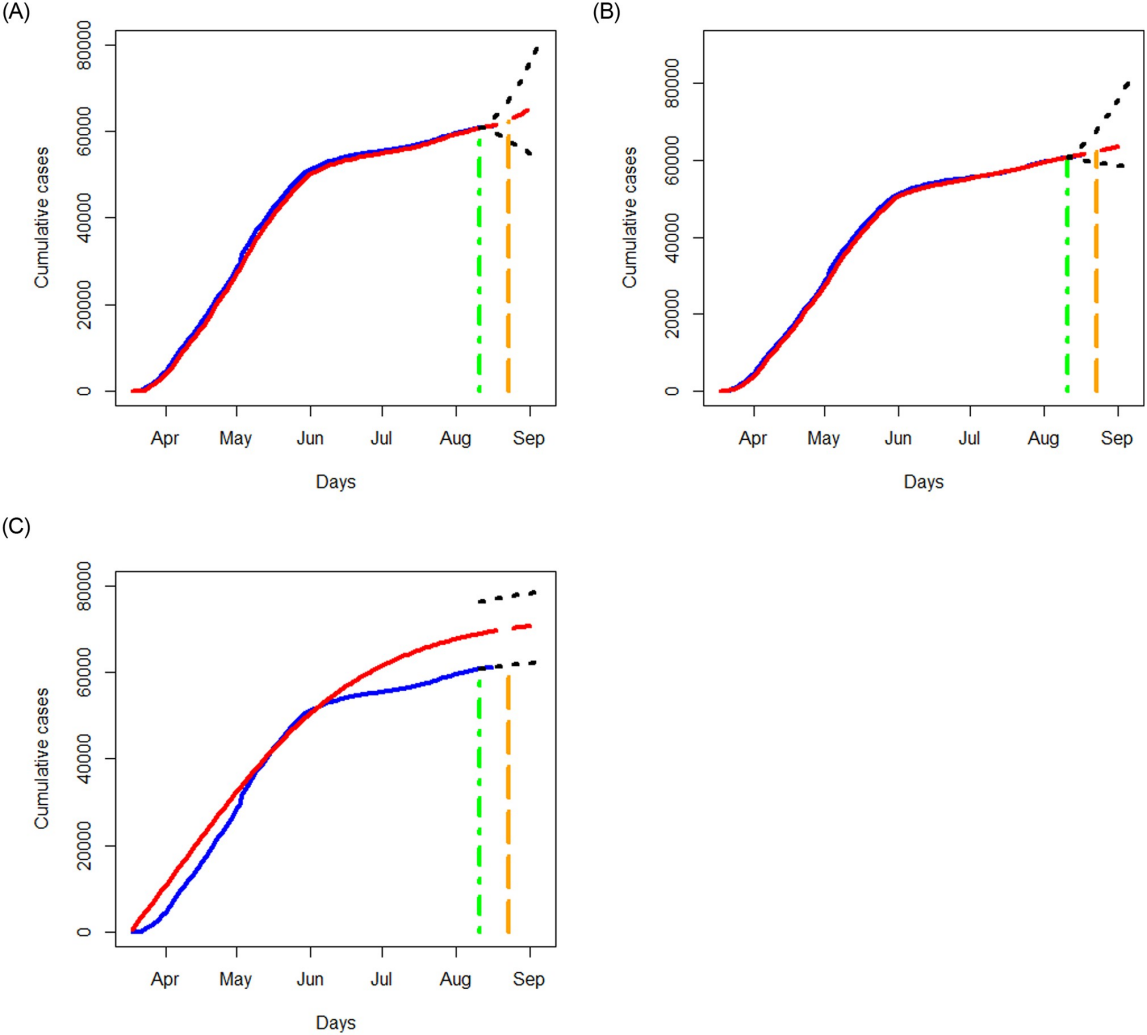
Analysis of cumulative numbers of infected cases with COVID-19 in Quebec, Canada using the STAR, NN and SIR models: Fitted values (in red) versus the reported cumulative infections (in blue). A red dashed curve represents the prediction for the next 25 days. Black dotted lines represent 95% prediction bands. The curves prior to the green vertical line are obtained for the training data; the red dashed curves show the predicted values where the green vertical lines indicate the start date of prediction. A region between green and orange dashed lines reflects a short term prediction, and a region after the orange dashed line shows a long term prediction.

**Fig 7:**
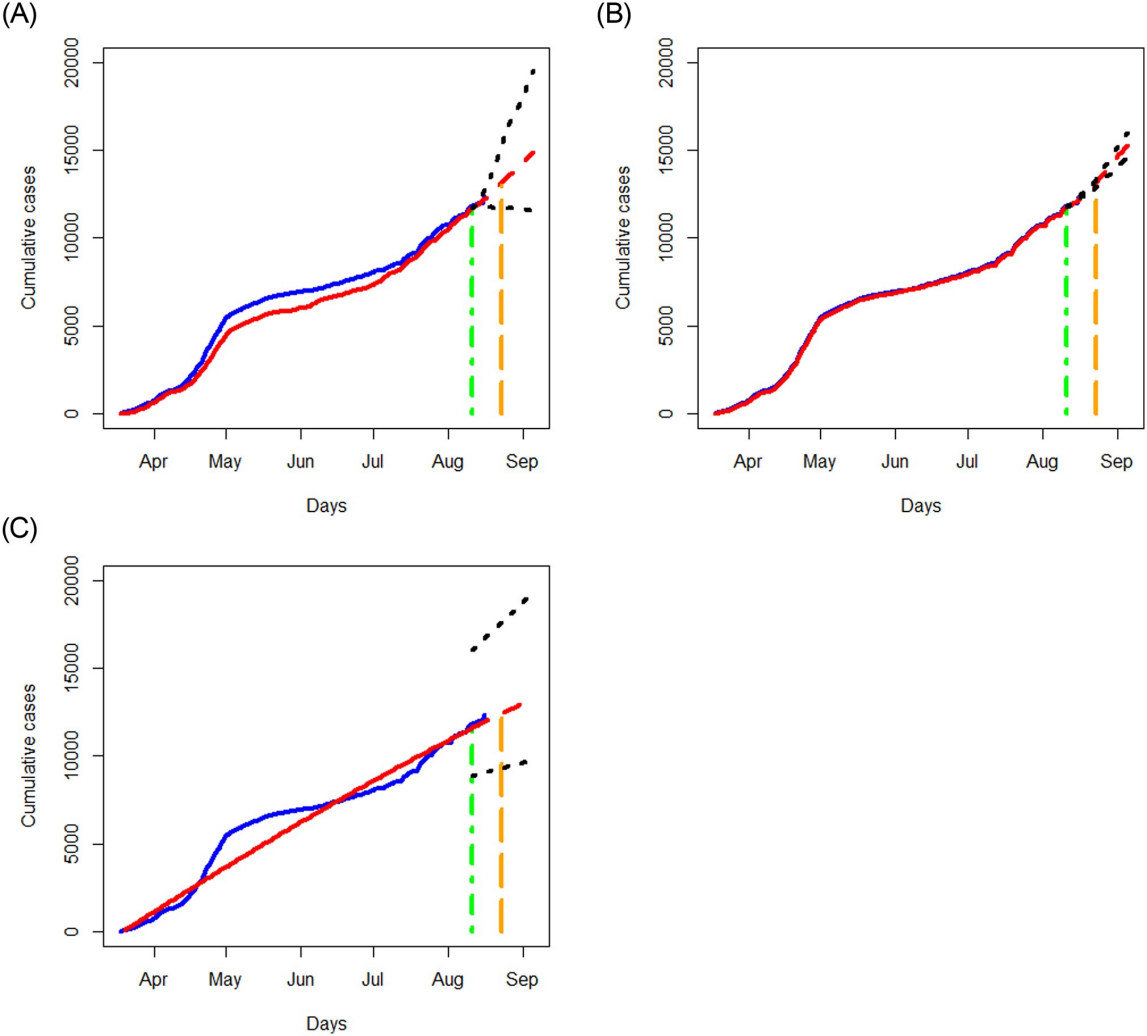
Analysis of cumulative numbers of infected cases with COVID-19 in Alberta, Canada using the STAR, NN and SIR models. All designations for the various curves are as described in Fig 6.

**Fig 8:**
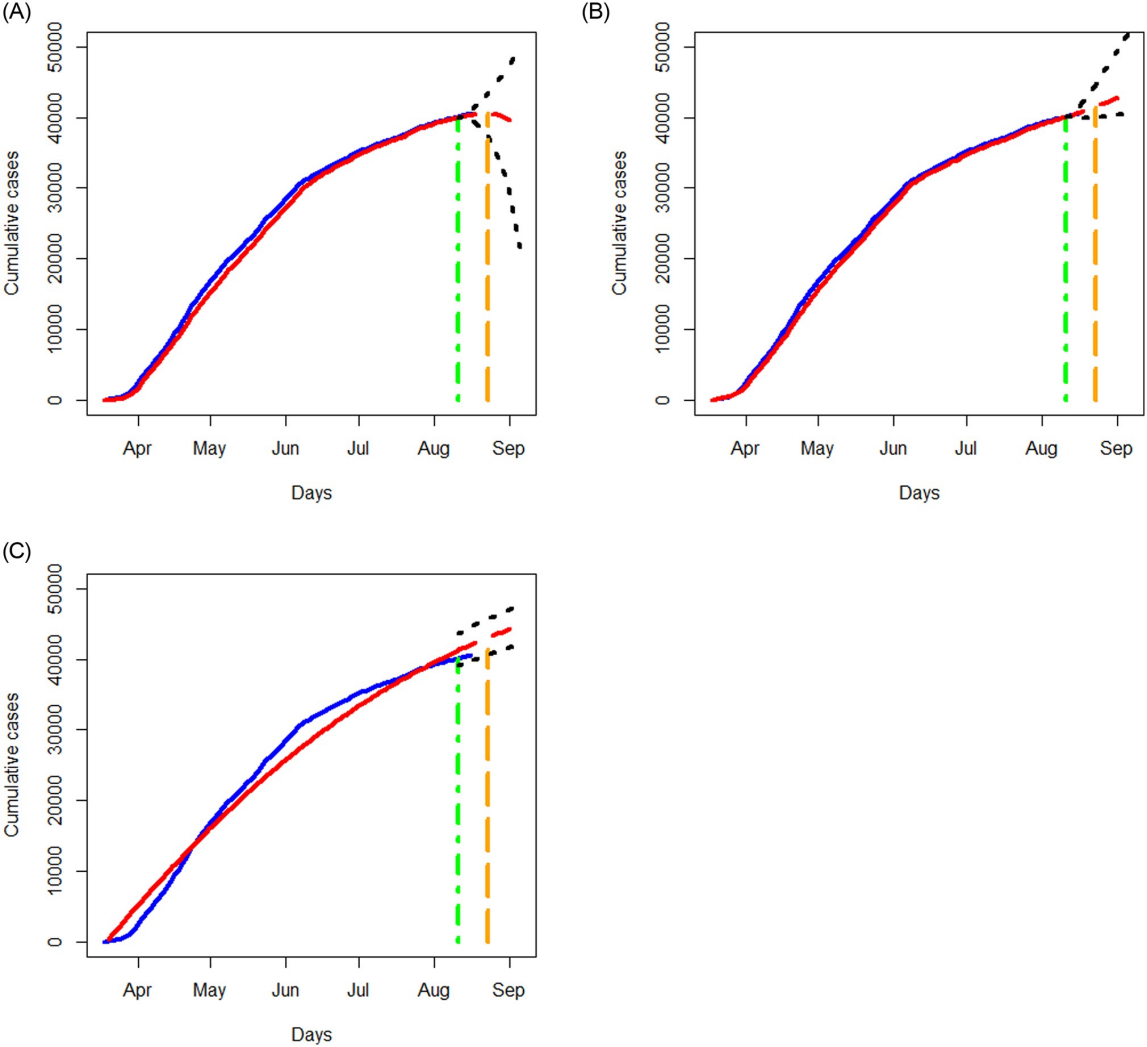
Analysis of cumulative numbers of infected cases with COVID-19 in Ontario, Canada using the STAR, NN and SIR models. All designations for the various curves are as described in Fig 6.

**Fig 9:**
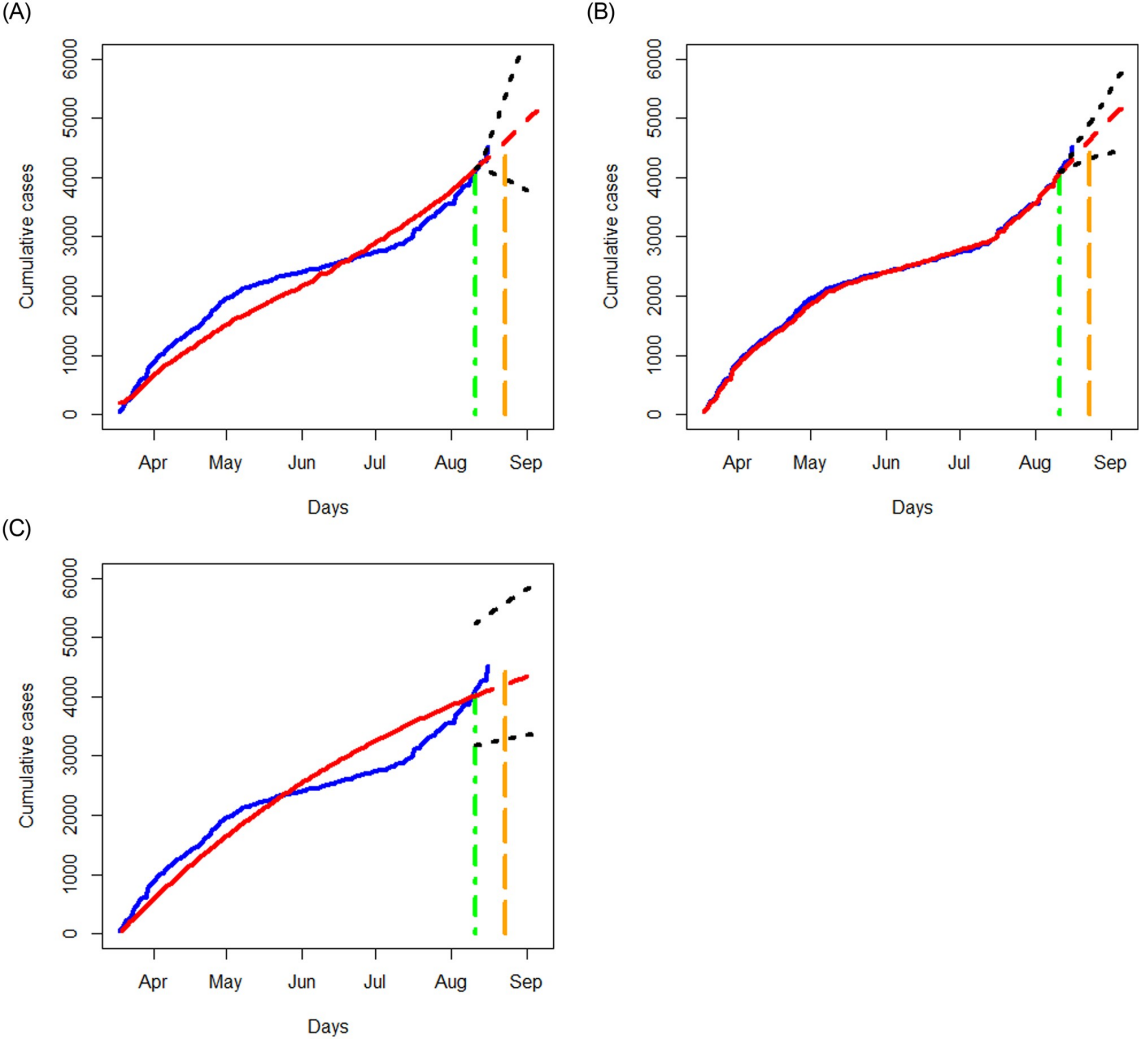
Analysis of cumulative numbers of infected cases with COVID-19 in British Columbia, Canada using the STAR, NN and SIR models. All designations for the various curves are as described in Fig 6.

We apply the three methods in Section 3.1 to examine the data of Quebec, Alberta, Ontario, and British Columbia separately, and respectively report in Figs 6–9 the results of the fitted values, predicted values and the associated 95% prediction intervals for the periods displayed in Table 2.

The NN method provides the most accurate fitted values than the STAR and SIR methods for the data in all the four provinces, and the SIR method tends to yield the worst fitted values. In terms of prediction, the NN method tends to have the smallest prediction region for the short term prediction. As expected, prediction errors for the three methods become bigger as the prediction period increases. The prediction trend for the short term agrees fairly well between those produced by the NN and STAR methods, but not with those obtained from the SIR model. Though there are disparities in the predicted results, all the methods predict an increasing trend for all the provinces except for the STAR method applied to the Ontario data.

While the SIR model does not seem to output better results than the NN method, it is advantageous in yielding some useful measures for describing the pandemic. For example, the basic reproduction number, defined as $R_{0}=\frac{\beta }{\gamma }$, represents the expected number of cases infected by one case in a population where all individuals are susceptible to infection. Basically, *R*_0_ is a simple yet informative measure to characterize the situation: “*R*_0_ > 1” means a coming exponential trend of the number of cases, “*R*_0_ = 1” implies a slow development of the pandemic, and “*R*_0_ < 1” suggests a dying down transmission of the virus. The larger value of *R*_0_, the harder to control the epidemic (e.g., Becker et al. 2006 [17]). With the estimates of *β* and *γ* produced by the SIR model, the *R*_0_ value for Ontario, British Columbia, Quebec, and Alberta are, respectively, given by 0.99, 0.98, 1.00, and 1.00. These estimates indicate a nonsevere pandemic situation in those provinces, thus in Canada, especially compared to the estimate, *R*_0_ = 5.7, evaluated for the initial period of the outbreak in Wuhan city, China [18].

Further, examining the reported number of cases, we notice that the increasing trend varies from province to province. The data in Quebec show an “elbow” shape with the “joint” appearing around June 1, 2020. The data in Ontario exhibit a somewhat similar shape to that of Quebec with a less conceivable “joint” being around June 10, 2020. The data in Alberta and British Columbia, on the contrary, display different patterns than those of Quebec and Ontario, yet they are somewhat similar in having two “changing” points. The data in Alberta and British Columbia show a steep increasing pattern until hitting May 1, 2020, then followed by a nearly flat shape until entering days around July 10, 2020 from which the increasing trend becomes sharp. While there are no obvious explanations why those patterns are different, some attributable factors include the time window of the containment measures (such as lockdown of cities) taken by the local governments, the population density, demographic structures, testing capacity, and healthcare facilities, as well as varying incubation periods (e.g., He et al. 2020 [19]).

### 3.3 Discussion of the three models

The numerical studies in Section 3.2 demonstrate that the three models output different results, though the differences can be negligible in some cases. These disparities basically pertain to the differences in the model assumptions and implementation procedures associated with those methods. The STAR model takes a parametric structure with the white noises assumed to have mean zero, and the associated parameters may be estimated by the least squares method. On the contrary, the NN method is model free and does not require an explicit function form to link the input and output variables. Instead, it calls for hidden layers with nodes linked by activation functions or linear functions, where different specification of those quantities facilitates various relationships between the input and output variables, and the associated weights are estimated based on the training data. Despite simple principles behind the SIR model, its validity relies on the invariance assumptions including a time-independent infection rate and a fixed size of the study population.

While those required conditions are generally difficult to be met or verified, applying those methods to analyze COVID-19 data may still reveal to some extent the progressive changes of the pandemic. For prediction over a short period, these methods provide fairly reasonable results and the NN method tends to outperform the STAR and SIR methods, evident from the good agreement between the predicted values and the reported numbers for the testing data. Unsurprisingly, the prediction ability of the methods for a long time window become less reliable, as shown by the increasing widths of the prediction intervals as the prediction period gets larger. These observations are consistent with the usual patterns of a reasonable prediction model: a long term prediction incurs more variation than a short term prediction (e.g., Chatfield 2001, p.478 [20]).

### 3.4 Analysis of the data in two states of USA

For comparison, in this subsection we employ the same methods discussed in Section 3.1 to analyze the COVID-19 data in two states of USA, New York and Texas, collected for the same period as the Canadian data discussed in Section 2.2 (i.e., March 18, 2020 to August 16, 2020).

**Fig 10:**
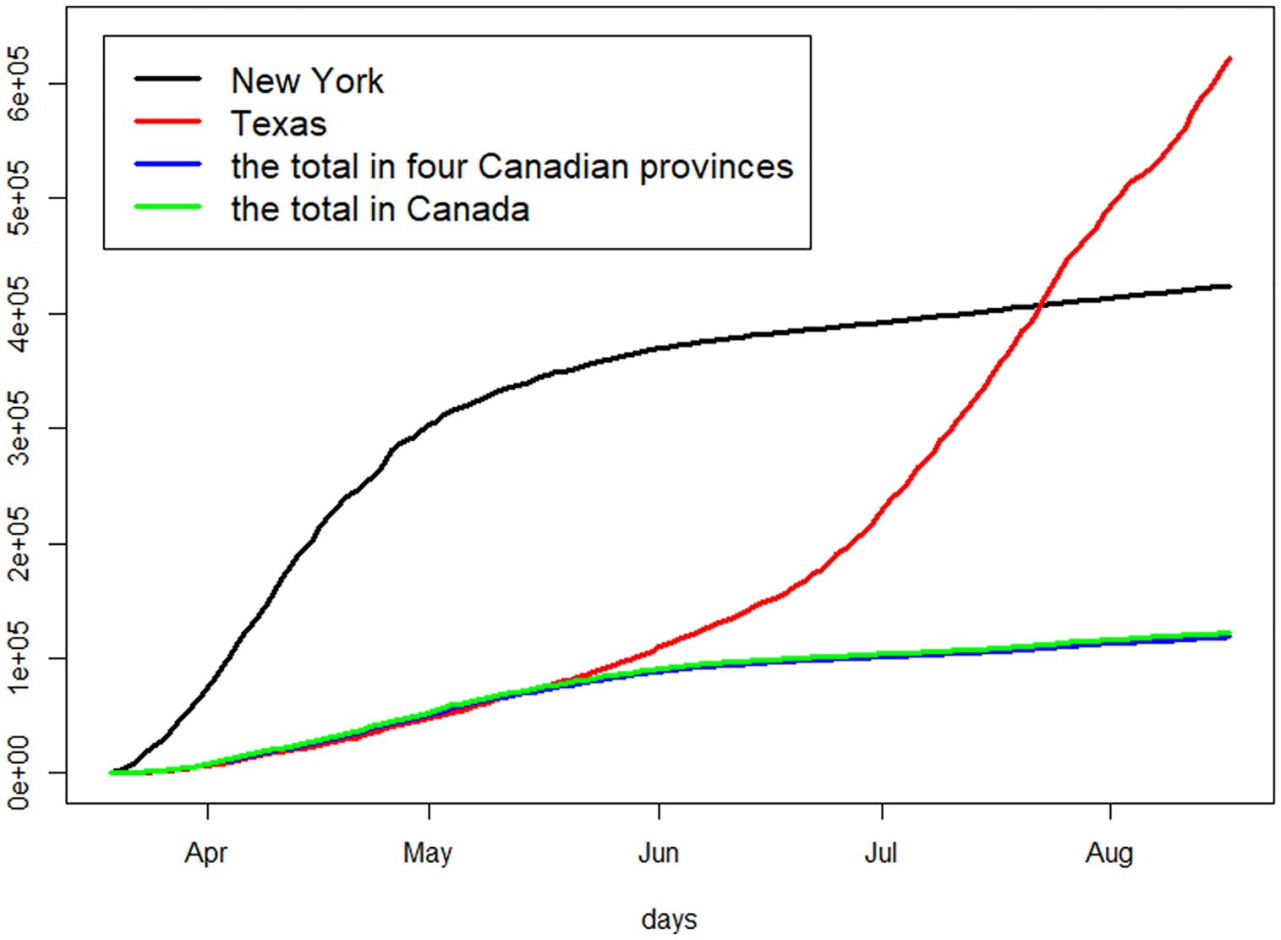
The cumulative numbers of reported infected cases: The total in the four Canadian provinces and the total in Canada, in comparison to those in New York and Texas, USA.

Fig 10 shows the cumulative number of infected cases of the two states, in contrast to the total of cumulative numbers of infections of the four Canadian provinces as well as that for entire Canada. New York state has a steeply increasing trend before June and then become relatively flat, whereas Texas shows a different pattern with a sharp upward trend. On the contrary, the total numbers of cases in Canada remain relatively low with a fairly flat trend.

**Fig 11:**
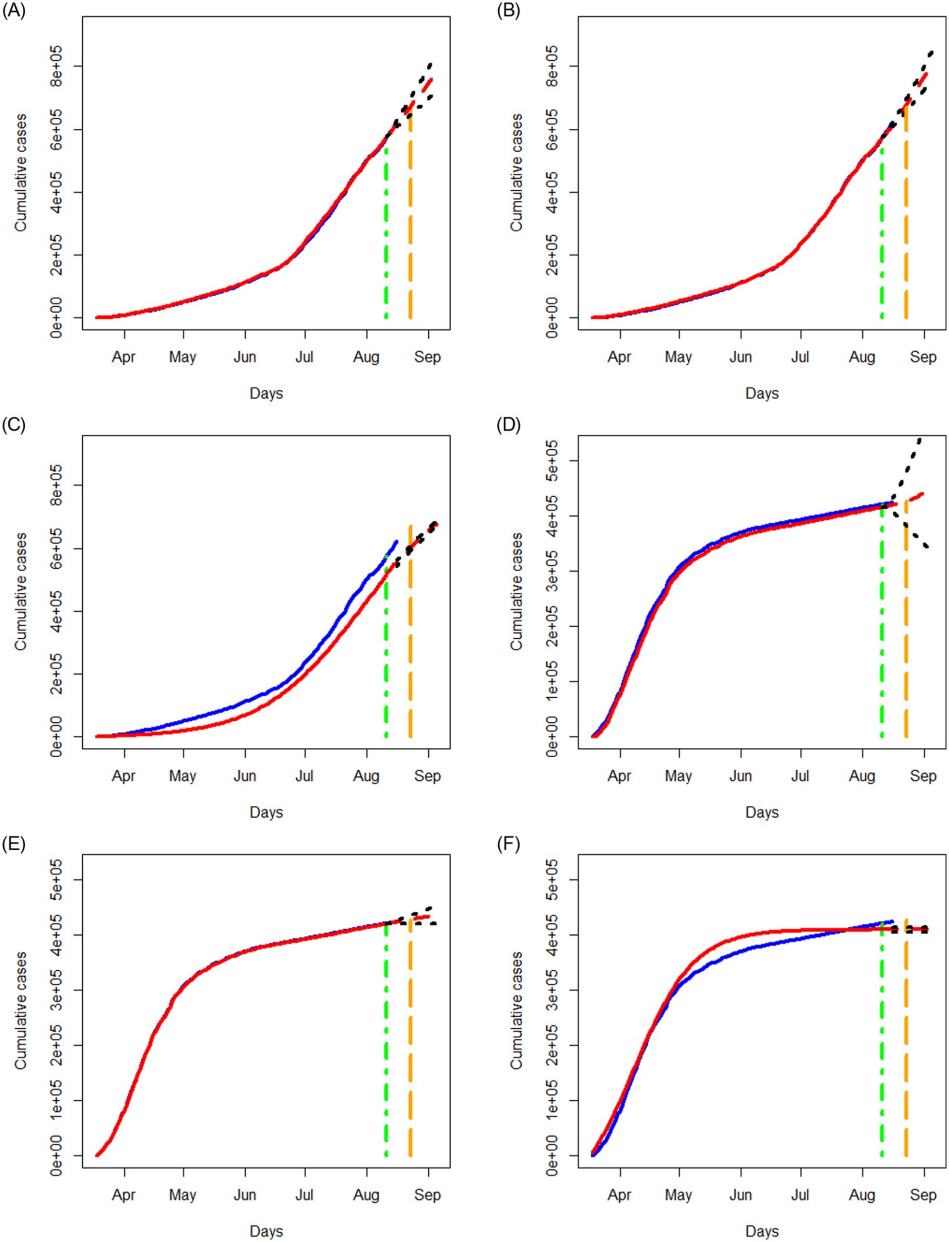
Analysis of cumulative numbers of infected cases with COVID-19 in Texas and New York state, USA using the STAR, NN and SIR models. All designations for the various curves are as described in Fig 6.

Similar to the analysis in Section 3.2, we apply the three methods to examine the data of New York state and Texas separately, where the data for the period of March 18, 2020 to August 11, 2020 are taken as the training data to build a prediction model, and the data for the period of August 12, 2020 to August 16, 2020 are taken as test data to assess the performance of prediction. In Fig 11, we report the prediction results for a short term period of August 17, 2020 to August 23, 2020 as well as for a longer period of August 24, 2020 to September 05, 2020, where we display the results of the fitted and predicted values, together with 95% prediction regions. Again, the NN method provides the best fit to the data with reasonably good prediction, and the SIR method tends to perform the worst. All the three methods predict a steeply increasing trend for the COVID-19 cases in Texas and a less sharp upward trend for New York state.

## 4 Discussion

In this paper, we investigate prediction of the development of COVID-19 in Canada using the STAR, NN, and SIR models. It needs to be emphasized that in building the prediction models, the associated model assumptions are not verified or not even realistic. For example, the SIR model requires no inbound or outbound infected travellers. It also assumes no asymptomatic cases, which is clearly untrue; combining a meta analysis with sensitivity analyses, He et al. [19] estimated that the asymptomatic rate was about 46%. From the epidemiological perspectives, it is important to incorporate asymptomatic infections (e.g., Moriarty et al. [21]) when building a prediction model in order to truthfully identify the number of infected cases. However, such information is unavailable for us to include in this study. A possible remedy is to conduct sensitivity analyses as outlined by He et al. [19], which is interesting to explore as a new project.

Another issue concerns the quality of data. The dataset itself may possibly have incorrect records for some days. For example, the dataset considered in this study is slightly different from the record in the JHU research dashboard (https://data.humdata.org/dataset/novel-coronavirus-2019-ncov-cases), and this may affect the validity of the prediction results as well. This article focuses the examination on time series data of reported daily infected cases. It is interesting to analyze other kinds of data such as daily recovered or daily deceased individuals. Furthermore, as data become richer and more accessible, it is useful to develop methods to study how the pandemic is associated with population-level characteristics as well as individual-level risk factors.
